# Pre-conception preparation at the antiphospholipid syndrome as way to improve reproductive health

**DOI:** 10.5195/cajgh.2013.104

**Published:** 2014-01-24

**Authors:** Gulyash Tanysheva, Saule Kabylova, Sholpan Kinayatova, Aizat Zhumazhanova

**Affiliations:** Semey State Medical University, Semey, Kazakhstan

**Keywords:** antiphospholipid syndrome, pre-conception

## Abstract

**Introduction:**

Reproductive health is characterized by the condition of the woman in association with the course of pregnancy and childbirth. In this case, the absence of disease plays a fundamental role. Unfortunately, conditions that can negatively impact reproductive health and cause deterioration of pregnancy and delivery outcomes are frequent in women of reproductive age. Antiphospholipid syndrome (APS) is one of the leading conditions that can negatively affect reproductive health and lead to various complications in pregnancy including fetal loss.

**Materials and methods:**

We assessed the effectiveness of pre-conception preparing, including traditional therapy of APS in conjunction with system enzyme therapy (SET) and plasmapheresis sessions. We conducted a study in two groups: women with APS and pre-conception preparing (n = 49) and the control group were women without pre-conception preparing (n = 46).

**Results:**

The effect of pre-conception preparing in women with APS was assessed by the course and outcome of pregnancy. The total number of women with complications of pregnancy were 39.1% lower in the study group compared to the control group. Risk of miscarriage in the basic group observed 68.7 % less frequently compared to the control group. The frequency of pre-eclampsia was 63.5 % less in the study group compared to the control group. We observed significantly lower rates of placental insufficiency in the study group and the difference in this parameter reached 65.2%. The risk of pre-term birth was 59.4 % lower in the study group compared to the control group.

**Conclusion:**

We concluded that pre-conception preparing in women with APS increases the possibility of physiological course pregnancy. Pre-conception preparing reduces the incidence of miscarriage, pre-term labor, and the development of pre-eclampsia, and placental insufficiency.

